# Lung Inflammation Predictors in Combined Immune Checkpoint-Inhibitor and Radiation Therapy—Proof-of-Concept Animal Study

**DOI:** 10.3390/biomedicines10051173

**Published:** 2022-05-19

**Authors:** Benjamin Spieler, Teresa M. Giret, Scott Welford, Tulasigeri M. Totiger, Ivaylo B. Mihaylov

**Affiliations:** Department of Radiation Oncology, Leonard M. Miller School of Medicine, University of Miami, 1475 NW 12th Ave., Suite 1500, Miami, FL 33136, USA; bxs737@med.miami.edu (B.S.); maidana.giret@gmail.com (T.M.G.); scott.welford@med.miami.edu (S.W.); tmt78@med.miami.edu (T.M.T.)

**Keywords:** lung, inflammation, pneumonitis, immunotherapy, radiotherapy, prediction, model, imaging, blood, bllod counts, cytokines, murine

## Abstract

Purpose: Combined radiotherapy (RT) and immune checkpoint-inhibitor (ICI) therapy can act synergistically to enhance tumor response beyond what either treatment can achieve alone. Alongside the revolutionary impact of ICIs on cancer therapy, life-threatening potential side effects, such as checkpoint-inhibitor-induced (CIP) pneumonitis, remain underreported and unpredictable. In this preclinical study, we hypothesized that routinely collected data such as imaging, blood counts, and blood cytokine levels can be utilized to build a model that predicts lung inflammation associated with combined RT/ICI therapy. Materials and Methods: This proof-of-concept investigational work was performed on Lewis lung carcinoma in a syngeneic murine model. Nineteen mice were used, four as untreated controls and the rest subjected to RT/ICI therapy. Tumors were implanted subcutaneously in both flanks and upon reaching volumes of ~200 mm^3^ the animals were imaged with both CT and MRI and blood was collected. Quantitative radiomics features were extracted from imaging of both lungs. The animals then received RT to the right flank tumor only with a regimen of three 8 Gy fractions (one fraction per day over 3 days) with PD-1 inhibitor administration delivered intraperitoneally after each daily RT fraction. Tumor volume evolution was followed until tumors reached the maximum size allowed by the Institutional Animal Care and Use Committee (IACUC). The animals were sacrificed, and lung tissues harvested for immunohistochemistry evaluation. Tissue biomarkers of lung inflammation (CD45) were tallied, and binary logistic regression analyses were performed to create models predictive of lung inflammation, incorporating pretreatment CT/MRI radiomics, blood counts, and blood cytokines. Results: The treated animal cohort was dichotomized by the median value of CD45 infiltration in the lungs. Four pretreatment radiomics features (3 CT features and 1 MRI feature) together with pre-treatment neutrophil-to-lymphocyte (NLR) ratio and pre-treatment granulocyte-macrophage colony-stimulating factor (GM-CSF) level correlated with dichotomized CD45 infiltration. Predictive models were created by combining radiomics with NLR and GM-CSF. Receiver operating characteristic (ROC) analyses of two-fold internal cross-validation indicated that the predictive model incorporating MR radiomics had an average area under the curve (AUC) of 0.834, while the model incorporating CT radiomics had an AUC of 0.787. Conclusions: Model building using quantitative imaging data, blood counts, and blood cytokines resulted in lung inflammation prediction models justifying the study hypothesis. The models yielded very-good-to-excellent AUCs of more than 0.78 on internal cross-validation analyses.

## 1. Introduction

Immune checkpoint inhibitors (ICIs), targeting programmed cell death protein-1 (PD-1) or programmed cell death protein ligand-1 (PD-L1), can provide lasting response and improve long-term survival in advanced non-small cell lung cancer (NSCLC) patients [1,2,3,4,5,6,7,8,9,10,11,12,13]. However, overall response rates to ICI monotherapy remain less than 50% and patients who do not respond can experience accelerated disease progression [14,15]. In comparison with cytotoxic chemotherapy, ICIs broadly offer an attractive side effect profile and are becoming a treatment of choice in various immunogenic cancers [16,17]. However, ICI-mediated immunomodulation can trigger adverse events in almost any organ system, with rash, vitiligo, gastrointestinal toxicities, pruritis, hyphophysitis, and pneumonitis most commonly observed [18].

Checkpoint-inhibitor-induced pneumonitis (CIP) is a potentially life-threatening adverse event seen most often among patients treated for NSCLC [19,20]. While life-threatening CIP is rare, in NSCLC patients, CIP can mimic conditions such as tumor progression or infection, complicating patient care. Most cases of CIP are mild and managed successfully in the outpatient environment, but inpatient management, long-term respiratory complications, or treatment-related deaths occur in ~20% of affected patients. Identification of patients at risk for CIP prior to immunotherapy could prevent significant morbidity, and discovery of signatures or methods for early CIP prediction represents an unmet clinical need in ICI cancer treatment [21].

Recently published studies suggest that systemic ICI therapy combined with local radiotherapy (RT) can result in enhanced systemic control of metastatic disease, surpassing the efficacy of ICI alone [16,22,23,24]. In the metastatic setting, ablative dose levels of focal RT have been theorized to potentiate ICI systemic effects through induction of an in situ vaccine, made possible by augmented antigen presentation and increased lymphoid cell trafficking due to radiation effects on the tumor microenvironment [23]. A logical but unintended consequence of an enhanced inflammatory state is an increase in number and severity of immune-related adverse events (irAEs) [25]. With the increasing use of ICI and RT, predictive models able to identify patients with elevated risk of irAEs, especially individuals without obvious predisposing factors, could offer clinical value.

In this study, it was hypothesized that lung inflammation after combined RT and ICI therapy is an observable phenomenon that can be predicted using pretreatment complete blood counts (CBCs), computed tomography (CT) or magnetic resonance imaging (MRI) radiomics [26,27,28,29], and liquid biopsy cytokines. The hypothesis was tested on a preclinical subcutaneous syngeneic murine lung tumor model, where CD45 (lymphocyte common antigen)-positive cells were used as a surrogate for lung inflammation [30,31,32,33]. CD45 is a pan lymphocyte stain. Therefore, it will indicate if there is an excess of lymphocytes accumulating in the lungs, i.e., inflammation. Based on that rationale, we decided to use CD45 as a biomarker for pneumonitis, following already published methodology.

## 2. Materials and Methods

In this proof-of-principle investigation, there was no a priori basis for sample size estimation. Details on the rationale for the number of animals used are presented elsewhere [34]. In brief, the principle of reduction in animal usage dictated an experimental design with 4 control and 15 treated (combined RT and ICI) mice. The study design and approach were approved by the Institutional Animal Care and Use Committee (IACUC, protocol: 17-214-ad02 EDR).

Lewis lung carcinoma (LLC) cells (Thermo Fisher Scientific, Inc., Waltham, MA, USA) were implanted subcutaneously on both flanks [35] in nineteen C57BL/6 mice. The cell line was cultured in medium Dulbecco’s Modified Eagle’s Medium (Gibco^®^; Thermo Fisher Scientific, Inc., Waltham, MA, USA), supplemented with 10% fetal bovine serum (Gibco^®^; Thermo Fisher Scientific Inc., Waltham, MA, USA) and 1% penicillin-streptomycin (HyClone; GE Healthcare Life Sciences, Logan, UT, USA). For subcutaneous implantation, a cell suspension of a density of 1 × 10^5^ cells/mL was prepared for each animal.

The mice (6–8 weeks old; 18–20 g) were purchased from Jackson Laboratory (Bar Harbor, ME, USA) and were housed at 22 ± 5 °C in a 12 h light/dark cycle and fed rodent chow and water ad libitum. Mice were subcutaneously inoculated with 100 µL LLC cell suspension (1 × 10^5^ cells/mL) under 2% isoflurane anesthesia. The skin was tented up, and the tumor cells were implanted under the skin in the dorsal regions of both left and right flanks. The tumor volume was measured several times per week by calipers, and calculated using the formula, [width^2^ (mm^2^) × length (mm)]/2 (volume of an ellipsoid).

**Figure 1:**
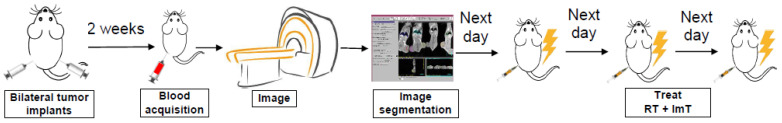
Study design: From left to right—bilateral tumors are implanted and 1–2 weeks later (tumor volumes ~200 mm^3^), blood is acquired, animals are CT- and MR-imaged, image segmentation is performed, and RT + ICI is started on the day after imaging. RT is delivered for three consecutive days. On each treatment day, tumors are irradiated with 8 Gy followed by PD-1 is administered through intraperitoneal injection. After tumor volumes reach the maximum allowed size, the animals are sacrificed, and lung tissue is acquired for immunohistochemistry.

The treated animals received a combined RT and ICI regimen according to a published and widely adopted schema [35]. Approximately ten days after tumor implantation, when the tumors reached ~214 mm^3^ on average (range from 99.8 mm^3^ to 437 mm^3^), noncontrast MRI and CT imaging were performed. Figure 1 outlines the experimental design and approximate timeline according to which the tumors were inoculated, imaged, and treated.

**Figure 2:**
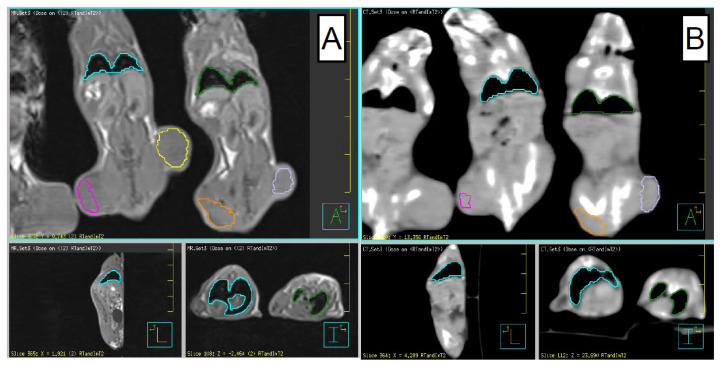
T1 3T MRI image (panel **A**) and CT image (panel **B**) of treated animals. The tumors on both flanks and the lungs are segmented for radiomics features extraction. The panels contain coronal (**top**), sagittal (**lower left**), and axial (**lower right**) views.

For CT scans, a resolution of 0.4 × 0.4 × 0.6 mm^3^ was used, while for the MR imaging, a T1 sequence with image resolution of 0.5 × 0.5 × 0.5 mm^3^ was employed. The CT hardware was 64-slice Siemens (Erlangen, Germany) Somatom Definition AS scanner, while the MRI hardware was 3T Siemens (Erlangen, Germany) TrioTim scanner. After imaging, the tumor volumes on both flanks as well as the bilateral lung volumes were digitally segmented in their entirety on both MRI (panel A) and CT (panel B), as represented in Figure 2 screen captures. For the CT images, semi-automatic lung segmentation was performed, using threshold levels set between 800 and 1200 Hounsfield Units. All segmentation slices were carefully reviewed for consistency of the contours. In all instances where the trachea was inadvertently included in the contours due to semi-automatic segmentation, it was manually removed since it is not part of the lung parenchyma. Lung segmentation in the MRI images was performed manually. In order to facilitate MRI contouring, the already segmented CT studies were co-registered with the MRI images, so that adequate lung representation was achieved.

Quantitative imaging features (radiomics) were then extracted from the imaging studies for tumors and lungs [34]. The average bilateral lung volume over the fifteen animals in the treatment cohort was 823.5 mm^3^, with the range being from 615.6 mm^3^ to 1093.2 mm^3^. This average lung volume corresponds to more than 8500 CT voxels, and in excess of 6500 MRI voxels, sufficient resolution for radiomics calculations to be performed on both imaging modalities.

In addition to the CT and the MRI imaging features, blood was collected on the day of imaging and CBCs and blood cytokine levels were acquired. Approximately 100–200 µL of blood were collected from each mouse and suspended in an Eppendorf tube pre-loaded with 10 µL of 0.5M EDTA. CBC flow analysis was processed using automated blood counter Element H5 (HESKA, Loveland, CO, USA). The data were collected from the blood counter and assessed for standard CBC components. Derived CBCs components included white blood cells (WBCs), neutrophils (N), lymphocytes (L), monocytes, eosinophils, basophils, red blood cells, etc. Ratios of those components were later used in downstream categorization.

After imaging, the animals were irradiated using a RadSource 2000 X-ray Irradiator cabinet (Rad Source Technologies, Buford, GA, USA) and organ-specific irradiation jigs (160 kVp, 25 mA, 0.5 mM Cu, 1.8 Gy min^−1^) under 2% isoflurane. The RT involved daily treatment for three consecutive days to the right flank only with daily doses of 8 Gy [35]. On each of these 3 days, RT was followed by intraperitoneal injections of 200 μg of PD-1mAb (BioXcell anti-mouse PD-1 (CD279)). When tumors reached the designated maximum volume per IACUC the animals were euthanized, and lung tissues were harvested. 

In order to measure the number of infiltrating lymphocytes into the lungs, the lungs were perfused with formaldehyde, processed and sectioned, and stained with an anti-CD45 antibody (Cat #14-0451-82, ThermoFisher Scientific, Waltham, MA, USA). The number of CD45 positive cells from three representative sections of lungs from each individual animal were quantified. The CD45 infiltration was used as a surrogate measure for lung inflammation and potential pneumonitis. The significant distance between the flank tumors and the lungs is evident in the coronal views in Figure 2. Furthermore, during flank irradiation, the animals were placed in specially constructed lead jig, covering the entire animal except the irradiated tumor. Therefore, the radiation dose to lungs due to internal scatter or shielding penetration was minimal and lung inflammation present in the treatment cohort was attributed to ICI.

Overall, 92 CT and 92 MRI radiomics features were extracted for the segmented bilateral lungs. The MRI radiomics were extracted after intensity normalization [36,37,38], which is a pre-processing step deemed appropriate for minimizing intersubject variance due to MRI scanner parameters. For each imaging modality, the features were divided into four groups—geometric features, first order histogram features, second-order joint probability features (e.g., co-occurrence matrices), and third-order joint probability features, originally described by our group [26]. The geometric, the first-, and the second-order radiomics features [29] are among the most commonly used features in the radiomics studies, while the third-order joint probability features were developed in-house [26]. Radiomics studies use a wide range of features which they report on—from couple dozens to couple thousands. The studies with fewer radiomics usually use simpler features which are easier to interpret, while the studies with a large number of features involve more convoluted ones, thereby being more difficult to understand and interpret. The selection of quantitative imaging features presented herein was based on their widespread use and easer interpretation. All of the radiomics utilized in this study were extracted with in-house developed software [26,39,40], interfaced with the Pinnacle (Philips Radiation Oncology Systems, Madison, WI, USA) treatment planning system.

The animals from the treatment cohort were divided into two groups (7 and 8 animals, respectively) based on the median CD45 values obtained from immunohistochemistry. After radiomics feature extraction, CBC counts, and cytokines acquisition, ANOVA (SPSS Statistics V.25 software package, IBM Corp., Armonk, NY, USA) analyses were performed on all variables and the dichotomized animal cohort based on CD45 infiltration. The imaging features, CBCs, and liquid biopsy cytokines with the highest statistical significance were selected. Furthermore, all selected variables were tested for correlation, where a Pearson correlation coefficient of 0.5 was used for cut-off. If any two significant variables were correlated with a coefficient larger than 0.5, one of the variables was removed from the pool used for subsequent model building. The remaining uncorrelated variables were subjected to binary logistic regression, aiming to model the prediction of lung inflammation. Logistic regression analysis predicts the outcome odds of a categorical variable based on one or more predictor variables. A categorical variable is one that can take on a limited number of values, levels, or categories, such as “valid” or “invalid”. A major advantage of logistic regression is that its predictions are always between 0 and 1. The most common and widely used form of logistic regression is binomial logistic regression, which predicts a single category or binary decision such as “pneumonitis” vs. “no pneumonitis.” The simplicity of this model dictated the split of CD45 infiltration around its median value, and its utilization for lung inflammation modeling.

Two-fold internal cross-validation was performed on the developed lung inflammation prediction model. In the first fold, 7 of the animals were utilized in model building, while the remaining 8 were used for model validation. In the second fold, the role of the two groups was reversed. Binary logistic modeling was performed with an SPSS analysis module. All binary logistic models included the independent imaging, CBC, and blood biomarker significant variables, as well as a model constant for better model fitting. 

## 3. Results

Since the tumors were implanted on the flanks of the animals, and only one of them was irradiated, the lungs were not directly affected by radiation—the large distance between the flank tumors and the lungs is evident on coronal views shown in Figure 2. Across the treatment cohort, the distance from the irradiated right flank tumor to the nearest lung tissue was ~2.5 cm, with the majority of the lung even further away from the irradiation field. Only the right flank tumor was irradiated with the rest of body shielded by a jig comprised by 2.0 mm of Pb. That amount of Pb shielding provides transmission of less than ~1.5%, since the half-value layer for Pb at 160 kVp is approximately 0.3 mm. Therefore, post-treatment lung inflammation was attributable to systemic effects rather than direct, local irradiation.

**Figure 3:**
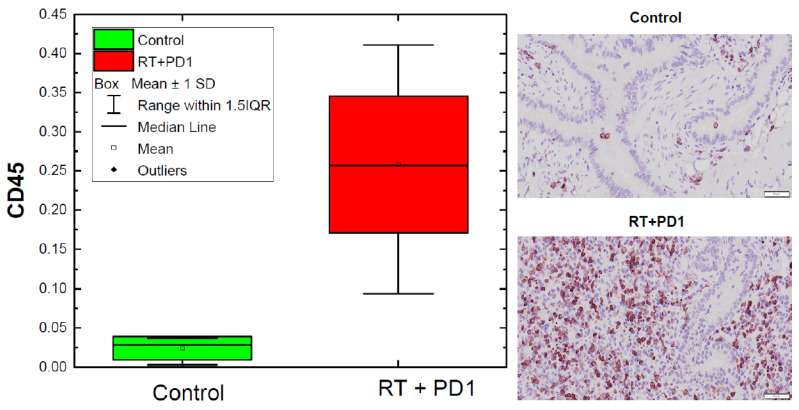
Immunohistochemistry analysis of CD45 cell infiltration in lung sections from control (**top right**) and treated with RT and ICI mice (**bottom right**). The box chart (**left**) demonstrates the quantitative analysis of CD45 distribution in the two cohorts.

Data from the immunohistochemistry analyses of paraffin-embedded mouse lung tissue using anti-CD45 antibody are presented in Figure 3 and Table 1. The table outlines the descriptive statistics of the observed CD45 infiltration. The second row in the table presents the data for the control (untreated animals), the third row presents the data for the entire treated cohort of 15 animals, while the fourth and fifth rows show the data for the low inflammation (i.e., below median from all treated animals) and high inflammation (i.e., above median from all treated animals) sub-groups. Based on the averages and the standard deviations for the low and high inflammation groups, the difference estimated with ANOVA analysis is significant at a *p*-value of 0.006. Figure 3 depicts that information in a graphical format. The difference between CD45 cell infiltration in lung sections from the control group (top right; magnification 20) and mice treated with RT and ICI (bottom right; magnification 20) can be clearly identified. The quantification on the box chart (left) demonstrates the statistical differences in CD45 among the control and the treated animals. Independent samples *t*-test analyses, performed with ANOVA, indicated a significant difference between the two means (control and treated) with a *p*-value of 0.0003. Furthermore, a large spread in the CD45 for the 15 treated animals, with minimum of 0.094, median of 0.26, and maximum of 0.411, is evident from the plot, indicating a substantial differential response which can be used as a surrogate for lung inflammation/pneumonitis. Close inspection of the range of values for the control and the treated cohorts indicates that the variability in CD45 infiltration in the treated group is several times larger than in the control. In Figure 3, there is marked difference between the staining of lung tissues between the two cohorts, and the spread of CD45 in the RT + ICI group (red bar on the plot) indicates a large differential response, i.e., different animals have different inflammation in the lung tissues, facilitated by the administration of ICI.

After CT and MRI radiomics extraction and ANOVA analyses, three uncorrelated CT radiomics features emerged as highly significant—average gray, histogram kurtosis, and co-occurrence matrix entropy. For MRI radiomics, only one feature was selected for correlation with CD45 infiltration—histogram kurtosis (details on the imaging features are provided in the Supplementary Materials, Table S1). 

Pretreatment CBC data are presented in Table 2. Minimum, maximum, average, and median values are reported for each parameter. The CBCs were derived from blood acquired prior to treatment. The only CBC component that correlated with increased lung inflammation was the neutrophil-to-lymphocyte ratio (NLR).

Table 3 presents data for liquid biopsy cytokines extracted from pretreatment blood serum. Similar to Table 2, minimum, maximum, average, and median values are reported. The only statistically significant cytokine associated with the observed CD45 difference was granulocyte-macrophage colony-stimulating factor (GM-CSF). 

Table 4 outlines the descriptive statistics for the CBC, cytokines, and the imaging features which were used for model generation. In addition, the *p*-values describing the differences between the corresponding features from the high and low inflammation groups are presented. Since the sample size is small and this is proof-of-principle study, the predictive model utilized uncorrelated features that differed up to *p*-values of up to 0.1.

**Figure 4:**
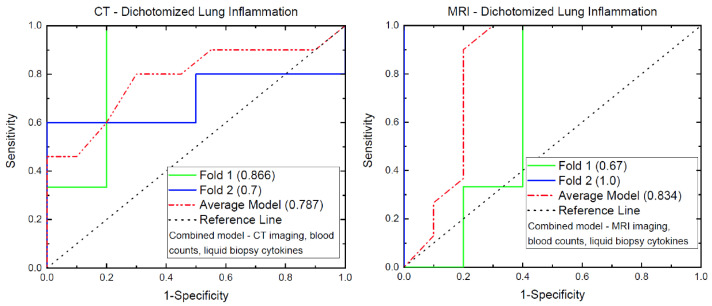
ROC analyses results from the modeling of lung inflammation. The corresponding AUCs for the two models are presented in the parenthesis in the caption. The green and the blue lines represent the ROCs from the two validation folds, while the red line is from the average of the two folds. The corresponding AUCs for the folds and the average are presented in the parenthesis.

The selected imaging, CBC, and cytokine variables were subjected to binary logistic regression for modeling the dichotomized CD45 infiltration distribution. Modeling was achieved using the binary logistic regression module in SPSS. The animal cohort was randomly split in two with seven animals in one group and eight animals in the other, so that internal cross-validation of the binary logistic regression model was possible. The results of that two-fold internal cross-validation are presented in Figure 4, where several AUCs are outlined. The CT model (left) consists of average gray values, histogram kurtosis, co-occurrence matrix entropy, NLR, and GM-CSF (details on the model values are provided in the Supplementary Materials, Table S2), while the MRI model (right) utilizes histogram kurtosis, NLR, and GM-CSF (details on the model values are provided in the Supplementary Materials, Table S3). The green and the blue lines in each panel represent the ROCs from the two validation folds, while the red line is the average from combining the two ROC curves. The corresponding AUCs for each fold and for the average are presented in the parenthesis. It is evident from the data that the combined model including the CT imaging features have AUCs over the folds larger than 0.7 and as high as 0.87, with an average of more than 0.75. The spread of the AUCs over the two folds with MRI quantitative imaging data is somewhat larger ranging from 0.67 to 1.0, but the average is also larger, exceeding 0.8.

## 4. Discussion

Very few studies have attempted to predict clinical pneumonitis after ICI treatment [41,42,43,44]. With expanded utilization of ICI therapies, identification and prediction of potentially lethal irAEs such as CIP gain clinical importance [21]. To our knowledge, this is the first preclinical investigation to study ICI-induced lung inflammation in a systematic fashion using quantitative imaging, blood counts, and blood biomarkers drawn from treatment-naïve animals. The variables identified can discriminate high versus low CD45 infiltration in murine lung tissue and have the potential to be used for prediction of ICI-induced pneumonitis in humans. The obtained average area-under-the-curve measurements for both CT and MRI imaging modalities were all larger than ~0.75, indicating good-to-very-good predictive power of the developed models [45]. Overinterpretation of these findings should be avoided. This proof-of-principle investigation was performed in a single murine strain using a single ICI agent in combination with extrathoracic RT. A limitation of our model is that ICI +RT is not commonly used in clinical practice (in extracranial disease) and a similar model using ICI without RT would be of advantage to strengthen the results. Our findings need to be generalized further in additional murine strains and for different immunotherapy agents. Lung inflammation herein was characterized by histopathology alone, while clinical lung pneumonitis is a diagnosis of exclusion informed by high-resolution CT and a constellation of clinical factors (shortness of breath, dry cough, low-grade fever, chest tightness, general malaise, etc.) That approach is not feasible in a murine experiment, so histopathology-based lung inflammation was used as a correlate. Despite these limitations, our findings suggest that pretreatment diagnostic imaging and common blood markers could be incorporated into a model predictive of human pneumonitis.

CD45 infiltration is not the only marker that can be applied as a surrogate for lung inflammation. An excellent review [46] of the cellular and molecular immune markers in lung cancer identifies additional surrogates for lung inflammation that merit investigation in future validation studies.

An elevated neutrophil count is known to stimulate tumor angiogenesis and contribute to disease progression or resistance to therapy, while fewer neutrophils and more lymphocytes (lower NLR) in the pretreatment state correlate with better treatment response [47,48]. According to one published clinical study [49], high pretreatment NLR and lymphocytopenia are associated with poor clinical outcomes and the reverse is true for lower NLR. Interestingly, the mean NLR was elevated by 25% in our murine cohort with low-level lung inflammation compared to the murine cohort with high-level lung inflammation. Taken together, these findings suggest that a high absolute peripheral lymphocyte count (low NLR pre-treatment) brings both a higher probability of RT/ICI response as well as a higher risk of inflammatory side effects such as ICI-induced pneumonitis.

## 5. Conclusions

Over the past decade, ICIs have revolutionized the battle against cancer and in combination with existing therapies have dramatically improved patient outcomes. However, utilization of the immune system to fight cancer can trigger clinically significant complications. Refinement in patient selection aided by models that predict treatment-related toxicity represents an important advance toward fully personalized ICI therapeutics. With the development of predictive models capable of discriminating subjects with elevated risk of irAEs, it may become possible to preemptively modify cancer management, intensifying or de-intensifying dose concentrations, switching agents based on side effect profiles, or selecting multi-agent treatment for patients with low risk of side effects.

Future directions in our research will include extension of this model to other murine strains and ICIs with intent to validate and refine a generalized group of imaging features, CBCs, and inflammatory cytokines that predict both type and severity of toxic response to ICI agents. Longer follow-up times will allow patterns of murine lung inflammation to mature and better reflect actual pneumonitis, making subsequent analyses more clinically relevant and translatable.

## Figures and Tables

**Table 1:** Descriptive statistics on CD45 infiltration obtained from the lung tissues through immunohistochemistry evaluation.

Group	Number Mice	Min	Max	Average	Median	Standard Deviation
Control	4	0.003	0.037	0.024	0.028	0.015
All treated	15	0.094	0.411	0.258	0.257	0.087
Low inflammation	7	0.094	0.217	0.176	0.195	0.041
High inflammation	8	0.263	0.411	0.324	0.327	0.055

**Table 2:** Descriptive statistics on the complete blood counts, derived from the blood samples acquired before treatment administration.

CBC Type	Min	Max	Average	Median
WBC (10^3^/µL)	0.98	7.95	5.246	5.38
Neu # (10^3^/µL)	0.37	3.09	1.823	1.82
Lym # (10^3^/µL)	0.53	4.43	2.994	3.07
Mon # (10^3^/µL)	0.05	0.42	0.249	0.26
Eos # (10^3^/µL)	0.02	0.28	0.115	0.11
Bas # (10^3^/µL)	0.01	0.12	0.064	0.06
Neu% (%)	26.4	42.2	34.493	33.8
Lym% (%)	48.1	65.5	56.86	57
Mon% (%)	2.2	8	4.94	5.1
Eos% (%)	0.8	5.7	2.4	2.2
Bas% (%)	0.5	1.8	1.3	1.4
RBC (10^6^/µL)	1.79	8.43	6.762	7.18
HGB (g/dL)	4	13.4	10.9	11.7
HCT (%)	8.8	42.4	34.07	36
MCV (fL)	48.5	52.7	50.38	50.2
MCH (pg)	15.6	22.6	16.43	15.9
MCHC (g/dL)	29.9	45.5	32.62	31.7
RDW-CV (%)	12.9	23.3	18.473	18.3
PLT (10^3^/µL)	184	1137	769.466	856
MPV (fL)	5.1	5.9	5.5133	5.6
NLR	0.404	0.873	0.618	0.641

#：Number.

**Table 3:** Descriptive statistics on the liquid biopsy cytokines derived from the blood serum derived from the pretreatment blood collection.

Cytokine	Min	Max	Average	Median
KC (A5)	33.345	380.18	138.93	95.16
TNF-α (A6)	3.77	29.59	12.75719	11.465
MCP-1 (A7)	232.235	2362.09	1190.526	1211.983
RANTES (A10)	41.355	41.355	41.355	41.355
IL-1β (B2)	4.87	29.22	10.61281	9.4725
IP-10 (B3)	82.44	512.41	320.8675	331.5525
GM-CSF (B4)	8.54	15.54	10.69313	9.905

**Table 4:** Descriptive statistics on significant blood counts, blood cytokines, and radiomics used in model building. The low/high notation denotes where the corresponding number is from the low or high inflammation group, based on the observed CD45 from Table 1. The last column outlines the calculated ANOVA *p*-value of the difference.

Feature	Min	Max	Average	Median	Standard Deviation	*p*-Value
NLR low/high	0.5/0.4	0.9/0.7	0.7/0.6	0.6/0.5	0.1/0.1	0.035
GM-CSF low/high	8.5/8.5	10.1/14.5	8.8/11.7	8.5/11.6	0.6/2.2	0.005
CT average gray low/high	265.1/255.9	291.2/309.9	278.4/289.9	277.4/292.2	9.0/15.3	0.104
CT histogram kurtosis low/high	1.9/2.2	3.0/6.6	2.6/3.8	2.7/3.3	0.4/1.7	0.093
CT co-occurrence matrix entropy low/high	11.9/12.2	12.3/12.5	12.1/12.3	12.2/12.3	0.1/0.1	0.012
MR histogram kurtosis low/high	1.8/2.1	10.4/6.8	6.1/3.7	7.4/3.3	3.3/1.6	0.091

## Data Availability

All supporting data is presented in the Supplementary Materials.

## Supplementary Materials

The following supporting information can be downloaded at: https://www.mdpi.com/article/10.3390/biomedicines10051173/s1, Table S1: All CBC, blood cytokines, CD45, CT, and MRI radiomics used in the regression modeling; Table S2: The model values of the parameters utilizing the CT imaging features in the binary logistic regression.; Table S3: The model values of the parameters utilizing the MRI imaging features in the binary logistic regression.
