# Supplementary material for: Lung Inflammation Predictors in Combined Immune Checkpoint-Inhibitor and Radiation Therapy—Proof-of-Concept Animal Study
Source: Biomedicines. 2022 May 19;10(5):1173. doi: 10.3390/biomedicines10051173 (PMC9138533; doi:10.3390/biomedicines10051173)
Supplement: Supplementary file 1 [file biomedicines-10-01173-s001.zip › biomedicines-1705419-supplementary.pdf]

Table S1. All CBC, blood cytokines, CD45, CT, and MRI radiomics used in the regression modeling.

Inflammation	CT Average Gray Value	CT Average Gray Value	CT Kurtosis 2D	CT Co-Occurrence Matrix Entropy	NLR	MR Kurtosis 2D	CD45
**high**	14.53	290.10	3.43	12.47	0.55	2.11	0.33
**high**	12.68	292.25	3.09	12.46	0.67	2.33	0.27
**high**	10.62	298.10	4.44	12.39	0.49	6.78	0.22
**high**	12.58	288.15	2.23	12.44	0.40	2.82	0.28
**high**	14.49	309.88	2.16	12.18	0.46	5.48	0.41
**high**	9.74	292.85	6.58	12.26	0.65	3.90	0.35
**high**	8.54	292.06	2.39	12.23	0.66	2.61	0.36
**high**	10.52	255.93	5.78	12.24	0.53	3.72	0.24
**high**	8.54	287.96	2.31	11.91	0.86	7.37	0.20
**low**	8.62	273.94	1.89	12.03	0.70	5.79	0.20
**low**	10.08	273.04	2.82	12.28	0.87	1.76	0.16
**low**	8.54	265.14	2.38	12.00	0.59	1.78	0.14
**low**	9.01	277.42	3.02	12.21	0.64	7.56	0.09
**low**	8.54	291.22	2.89	12.26	0.64	10.37	0.19
**low**	8.54	279.77	2.71	12.26	0.54	8.09	0.21
**low**	14.53	290.10	3.43	12.47	0.55	2.11	0.33

Table S2. The model values of the parameters utilizing the CT imaging features in the binary logistic regression.

Parameter	Model Value
GM-CSF	0.86
CT Average Gray Value	0.86
CT Kurtosis 2D	0.09
CT Co- Occurrence Matrix	147.27
NLR	26.59
Constant	-2077.89

Table S3. The model values of the parameters utilizing the MRI imaging features in the binary logistic regression.

Parameter	Model Value
GM-CSF	1.85
MR Kurtosis 2D	-3.03
NLR	-37.42
Constant	21.13
